# Gender-Specific Differences in Concentrations of Biochemical Parameters in Persons over the Age of 90

**DOI:** 10.3390/ijerph16111915

**Published:** 2019-05-30

**Authors:** Paulina Zabielska, Sylwia Wieder-Huszla, Izabela Gutowska, Anna Lubkowska, Anna Knyszyńska, Anna Jurczak

**Affiliations:** 1 Department of Social Medicine and Public Health, Chair of Social Medicine, Pomeranian Medical University in Szczecin, Żołnierska Str. 48, 71-210 Szczecin, Poland; paulina.zabielska@pum.edu.pl; 2 Department of Clinical Nursing, Pomeranian Medical University in Szczecin, Żołnierska Str. 48, 71-210 Szczecin, Poland; anna.jurczak@pum.edu.pl; 3 Department of Human Nutrition and Metabolomics, Pomeranian Medical University in Szczecin, Broniewskiego Str. 24, 71-460 Szczecin, Poland; izagut@poczta.onet.pl; 4 Department of Functional Diagnostics and Physical Medicine, Pomeranian Medical University in Szczecin, Żołnierska Str. 54, 71-210 Szczecin, Poland; anna.lubkowska@pum.edu.pl (A.L.); anna.knyszynska@pum.edu.pl (A.K.)

**Keywords:** longevity, gender differences, inflammation

## Abstract

The ageing process is a phenomenon leading to an emergence of a web of interrelated biological processes producing significant changes in the human body. The aim of the paper was to analyse gender-specific differences with regard to selected biochemical parameters in longevity. The study was conducted in Poland in 2017. The study population comprised of long-lived individuals, both living at home and staying at residential care homes. There were 90 people of both genders (69 women and 21 men) aged between 90 and 103 years (mean = 92.36; SD = 2.98). The biochemical markers of longevity in the studied patients were identified by assessing their pro-inflammatory and anti-inflammatory status in various metabolic aspects. The analysis of biochemical variables revealed numerous statistically significant differences, both in the study group as a whole and between the genders. The ageing process is extremely complex, but understanding it will allow for a real intervention in life extension. Research into the processes involved in ageing and longevity will enable a better understanding of the mechanisms responsible for them, and to enjoy those processes in good health.

## 1. Introduction

In recent years, with the progress of civilisation and breakthroughs in science, life expectancy has been increasing around the world. This phenomenon may be attributed mainly to the development of new medical technologies and more effective treatment methods, but also to the advent of healthy lifestyles and active ageing measures which have been widely promoted since late 20th century [1]. Over the past few decades, in many countries there has been a marked increase in the number of longevous people, aged 90 years and more (especially centenarians), and in the coming years this population is expected to grow. Centenarians as a group account for the most spectacular growth, also within the Polish society. According to Statistics Poland (GUS), in 2007 there were 1390 centenarians in 2013–3196, and according to the forecasts by 2035 the number of people aged 100 years and over is expected to exceed nine thousand. Further, the nonagenarian population in Poland is projected to nearly double by 2035 [2]. Ageing, followed by old age, is one of the least known stages of the human life cycle. Changes taking place in that period occur in many dimensions. Therefore, it makes sense to engage in a multi-faceted analysis of the ageing process, carried out by interdisciplinary teams with expertise in biomedical science as well as social studies [3]. The exceedingly individual nature of this process cannot be denied, and the length of human life varies widely, not only across individuals and populations, but also genders. On a global scale, women live on average 6 years longer than men, and whose ageing process generally tends to be accelerated [4]. 

As life expectancy increases in developed countries, there are some serious risks related to the growing numbers of elderly people with cognitive impairment and dementia in the overall population. Such a situation is associated with a decline in physical activity, brain structural integrity and secretion of growth factors, such as the growth hormone (GH) and insulin-like growth factor-1 (IGF-I) [5]. The guidelines on the use of GH take into account patient age and gender. Women require higher doses of the hormone used over a longer duration to obtain the same IGF-1 response as men. For elderly patients, GH dosage is lower, and the period of therapy is longer than in the younger population [6].

Age is directly associated with the incidence of cardiovascular incidents, where elevated levels of homocysteine are a predictive factor. Therefore, measuring the level of that amino acid is vital in the prevention and treatment of cardiovascular disease [7,8]. As an additional risk factor in post-menopausal women, the decline in oestrogen levels impairs the regenerative capacity of the cardiovascular system, and at a later stage leads to age-related microvascular rarefaction [9].

Reduced physical activity in post-menopausal women contributes to persistent inflammation, which is perceived as endothelial dysfunction and vascular alterations [10]. It is also associated with excessive accumulation of adipose tissue, which secretes elevated amounts of pro-inflammatory cytokines, including tumour necrosis factor alpha (TNF-α), interleukin-1 beta (IL-1β) and interleukin-6 (IL-6) [9]. Studies have also indicated a correlation between the levels of pro-inflammatory cytokines and symptoms of depression in the elderly population. Inflammation plays a key role in the onset and progression of atherosclerosis. Therefore, the measurement of inflammatory markers in blood serum is useful in risk assessment for cardiovascular disease. The most commonly used inflammatory markers include: IL-6, C-reactive protein (CRP) and TNF-α. There is little data on the differences in IL-6, CRP and TNF-α levels between men and women [10]. Elevated CRP was also observed to be associated with a depressed mood in people over the age of 65 [11]. Elevated blood levels of pro-inflammatory markers, such as CRP and IL-6, are found in patients with chronic obstructive pulmonary disease (COPD) [12].

Recent studies demonstrated that adipose tissue serves as a source of additional molecules that integrate metabolism, immune response and normal vascular function. Leptin is structurally similar to IL-6 which is responsible for some physiological actions, such as food intake and angiogenesis, at the same time sustaining a proinflammatory environment. Likewise, resistin modulates several metabolic and inflammatory pathways and plays an important role in the development of atherosclerosis. On the other hand, adiponectin features prominently in the maintenance of endothelial function, displaying anti-inflammatory, and antiatherogenic properties [9,13]. In the elderly, a high IL-6 level is associated with increased mortality, mainly due to neoplastic processes [14].

The circulating inflammatory markers related to CRP and fibrinogen are associated with the risk of ischemic heart disease and stroke [15,16]. Fibrinogen is a marker of both hypercoagulability and inflammation. The role of subclinical inflammation in the development of atherosclerosis and as a contributing factor in acute coronary episodes has been proven long ago [17]. Available research findings also show fibrinogen to be a stronger risk factor in the development of coronary heart disease than cholesterol. Fibrinogen levels are correlated with the severity of calcification and progression of lesions in coronary arteries. High fibrinogen levels in people with unstable coronary heart disease enhance the risk of a heart attack and death, while persistent elevated levels after a heart attack are an unfavourable prognostic factor [18]. Increased concentrations of fibrinogen are observed in stable chronic obstructive pulmonary disease (COPD), and during the acute stage of the disease, it is accompanied by an upswing in serum levels of IL-6. Therefore, it is highly likely that increased levels of the above parameters are associated with accelerated development of vascular atherosclerosis in this patient group [19].

Gender and gender-specific quantitative and qualitative differences in hormone levels unquestionably impact on the function of the body as a whole, and also determine its response to various stimuli. On the other hand, ageing and the associated disorders in hormone synthesis, particularly with respect to sex hormones, affect metabolism as a whole. Having regard to the above, the aim defined for our study was to analyse gender-specific differences with regard to selected biochemical parameters in longevity.

## 2. Materials and Methods

### 2.1. The Study Group

The study was conducted in Poland in 2017, in two voivodeships (provinces)—West Pomerania (Zachodniopomorskie) and Masovia (Mazowieckie). The people inhabiting Masovia are for the most part indigenous to Poland, whereas West Pomerania is characterised by a population with a migrant background, due to the resettlements after 1945. The study population comprised long-lived individuals, both living at home and staying at residential care homes. There were 90 people of both genders (69 women and 21 men) aged between 90 and 103 years (mean = 92.36; SD = 2.98). Women accounted for 76.7% of the studied population (mean age = 92.59; SD = 3.15), and men 23.3% (mean age = 91.57; SD = 2.20). 

Fasting blood samples were collected from each patient (at least 8 h since the last meal): 7.5 mL of blood from an available vein into a Vacutainer clot activator tube. The blood was centrifuged (1500 *g*/15 min/room temperature), and the obtained serum was used to determine the levels of selected biochemical parameters.

All patients were thoroughly informed about the scope and objectives of the study and gave their written consent for participation. The study was approved by the Bioethics Committee of the Pomeranian Medical University (no. KB-0012/47/16).

The biochemical markers of longevity in patients were identified by assessing their pro-inflammatory and anti-inflammatory status in various metabolic aspects:

- determination of concentrations of proinflammatory (IL-6, IL-1, TNF-α, CRP, fibrinogen) and anti-inflammatory (L-10, TGF-β1) factors;
- determination of signalling pathway factors: insulin, IGF-1 (insulin-like growth factor 1);
- determination of the concentration of adipokines, hormones produces by adipose tissue: adiponectin, leptin and resistin;
- evaluation of hormone levels: growth hormone–releasing hormone (GHRH, somatocrinin), thyroid-stimulating hormone (TSH, thyrotropin), growth hormone (GH), cortisol, gonadotropins: follicle-stimulating hormone (FSH) and luteinizing hormone (LH);
- determination of activity of antioxidant enzymes and glutathione (reduced and oxidised forms) in plasma and in isolated red blood cells.

### 2.2. Statistical Analysis

Statistical analyses were performed using the IBM SPSS Statistics v.21 suite (IBM, Armonk, NY, USA). To compare groups in terms of the distribution of nominal variables, Pearson’s chi-squared test was employed, and the comparison of means in two independent groups was used for quantitative variables. Due to the significant difference in the size of the groups subject to comparison, the non-parametric Mann-Whitney U test was chosen. A multiple linear regression model was also used. The statistical significance cutoff was set at *p* < 0.05, and *p* < 0.1 was adopted as a marker of a statistical trend. The tables were made in APA format.

## 3. Results

Following the analysis of the results of biochemical examinations in the study group taking into account the patients’ sex, no statistically significant differences were found in the selected biochemical parameters (Table 1). Differences between men and women of borderline statistical significance were found only in two of the analysed blood serum parameters. The average levels of HGH (human growth hormone) and TNF-α were higher in women (*p* = 0.067 and *p* = 0.071).

The Spearman rank correlation analysis of biochemical variables revealed numerous statistically significant differences, both in the study group as a whole and between the genders. The tables present only such correlations for which significance was found in the general group and/or one of the genders (Table 2, Table 3, Table 4, Table 5, Table 6 and Table 7).

With respect to insulin, its serum levels are statistically negatively correlated with the concentrations of cortisol, HGH and adiponectin in the general group, and none of these relationships were found in the women’s group. Women are however, predisposed to a positive and statistically significant correlation between serum levels of insulin and IL-1α (Table 2).

With regard to serum CRP levels, a higher number of statistically significant correlations was found in women. Importantly, a decline in LH and FSH levels in older women was observed to cause a significant increase in CRP. In turn, serum HGH levels in older men exhibit a higher number of significant correlations with the biochemical parameters selected for analysis (LH, IGF-1, GHRH, adiponectin), all of them positive. The correlation between serum HGH and GHRH levels (growth hormone-releasing hormone) seems to be particularly significant in women (Table 3), as they were also found to have a significant positive dependence between GHRH and adiponectin (Table 5).

The decline in serum LH levels in old age is accompanied by decreased in FSH levels, and this correlation was observed both among women and men. Meanwhile, women are predisposed to a significant positive relationship between LH levels and the concentrations of antioxidant enzymes (GPx, CAT, SOD, GST) and antioxidants (GSH), in addition to a negative relationship between the level of LH and those of resistin and IL-6. With respect to homocysteine, all statistically significant correlations in the analysis were positive and found in both sexes (Table 4).

Correlation analysis of IL-6 levels and other parameters included in the analysis again demonstrated that women are more predisposed to a significant relationship between the studied parameters. Positive correlations were found for IL-6 versus SOD, GST and homocysteine, and negative correlations – for IL-6 versus LH and IL-1β (Table 6).

Further, in the case of significant correlations between FSH (follicle-stimulating hormone) and other variables in the analysis, female gender rather than male seems to be the determining factor. With regard to other variables (GST, GSSG, R-GSSG), in the women’s group, statistically significant relationships were observed more frequently than in the men’s group. The strength of relationship for statistically significant results was similar in the representatives of both genders (Table 7).

## 4. Discussion

Ageing is a physiological process affecting all people. Regardless of biological and environmental changes taking place, comprehensive research may help us expand our knowledge on the mechanisms influencing longevity. Understanding the processes taking place in old age would make it possible to develop a specific antidote to ageing. The ageing process is a phenomenon leading to an emergence of a web of interrelated biological processes producing significant changes in the human body. The scientific exploration of bodily functions in old age is greatly furthered by the study of biological parameters, lending themselves to a quantitative and qualitative assessment of various conditions and phenomena presenting themselves in the ageing body. The biological factors subject to exploration are a valuable and constantly evolving source of knowledge on longevity and methods for diagnosing, treating and preventing diseases. The complex mechanisms driving the changes related to body ageing take place in cells and tissues in an imprecise and inconsistent manner. The ageing process may be delayed by lifestyle, including physical exercise, nutrition and the surrounding environment.

The available literature discussing research into the impact of changes in selected biochemical factors on long-living people is rather scarce. This area of medical science is still being studied by scholars around the world and keeps expanding to include more and more new longevity research methods. Our study was aimed at identifying changes which may occur in selected biochemical factors in very old age. Findings from these studies may help establish the molecular profile of people aged over 80 years. 

### 4.1. Influence of Leptin and Adiponectin Levels on Longevity

In a study by Pareja-Galeano et al. [19] devoted to leptin and adiponectin levels in longevity, higher concentrations of the former were observed (*p* < 0.001) in the group of centenarians compared to the control group made up of elderly individuals, whereas no statistically significant differences were observed for adiponectin levels. It is suggested that further research should be conducted to explain the influence of high levels of proteins secreted by adipocytes on health and vitality in old age [20]. Our study confirmed that there was no correlation between adiponectin and gender in the study population (*p* = 0.663). On the other hand, the analysis of 80 obese individuals performed by Selthofer-Relatić et al., where the mean age for men was 59.47 ± 7.60 years, and for women 64.10 ± 7.59 years, demonstrated that women have a substantially higher ratio of leptin to adiponectin than men [21]. Further research in this scope is needed.

### 4.2. Markers of Inflammation in Longevity

The ageing process is directly related to the immune and inflammatory function, which has been confirmed by many scholars [22,23,24]. Hurme et al. [25] presented substantial evidence pointing to the relationship between the rate of inflammation and human longevity. Elevated concentrations of inflammation markers, i.e., TNF-α and IL-6, may lead to increased mortality rates among the elderly, thus reducing survival in this age group [22,23,24]. This was confirmed by Bonafe et al. [26] who demonstrated that people genetically predisposed to produce high levels of IL-6 during ageing are less likely to reach the extreme limits of the human life span, which proves that low IL-6 levels have a positive impact on longevity. Researchers were also able to demonstrate that in women, there is no correlation between high IL-6 and old-age survival, unlike in men, which may result from the inhibiting effect of oestrogens on the expression of the IL-6 gene [26]. Our own study, however, did not reveal a dependence between IL-6 and gender (*p* = 0.806). The authors, Chen CJ et al. and Prather AA et al. analyzing younger populations than the population described in our study, assert that there are differences in IL-6 levels between sexes [27,28].

Ruan et al. in their study attempted to identify the biomarkers of human ageing. They found a correlation between the activity of antioxidant enzymes SOD (*p* = 0.003), POD (*p* = 0.004), GSH-px (*p* = 0.018) and pro-inflammatory cytokine IL-6 (*p* < 0.001) with the age of the study participants. The other parameters included in the study (IL-10, IL-1β) were not significantly correlated with age [29]. Our own findings also confirmed positive correlations between the level of IL-6 and SOD, GST (Table 6).

**Figure 1:**
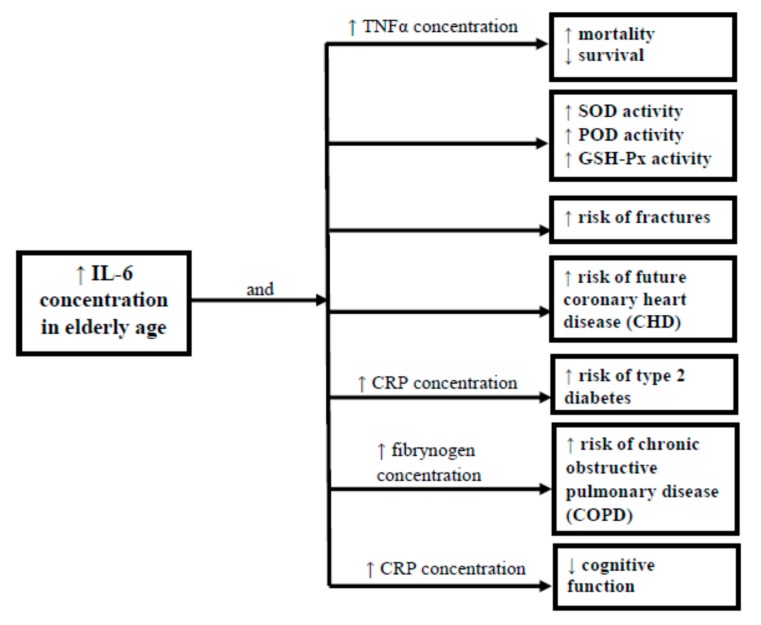
Relations between biochemical parameters and the supposed consequences in health and longevity.

Cauleyetal. [30], studying a population representing the 70–79 age group, proved that elevated inflammatory markers are associated with an increased risk of fractures. Moreover, Rodondi et al. [31] demonstrated that IL-6 and AAI (ankle-arm index) are associated with future coronary heart disease (CHD) events beyond traditional risk factors and modestly improverisk predictions in older adults [31]. Other studies conducted in a senior population revealed a relationship between elevated levels of IL-6, CRP and diminished cognitive function [32], as well as an increased risk of type 2 diabetes [33]. Moreover, increased serum IL-6 is more strongly, than other inflammatory markers (CRP, TNF-α), associated with the risk of disease, disability, and mortality [34]. Almeida et al. [35] found a relationship between frailty in old age and increased levels on CRP, while Tiainen et al. [36] pointed to a correlation between chronic inflammation and that condition. On the other hand, the team of Liu et al. [37] in their study of a group aged 70–84 failed to identify any relationship between these variables, while a study by Lim et al. in a group of nonagenarians found lower levels of IL-6 and THFR1 and higher levels of IGF-1 in the subgroup with the highest cognitive status [38]. All this points to the complexity of the processes taking place and the interaction between the biochemical parameters in ageing individuals, and that is why it seems necessary to continue research, taking advantage of already gathered data [39], particularly in groups of longevous individuals (Figure 1).

### 4.3. Gene Expressions and Longevity

**Figure 2:**
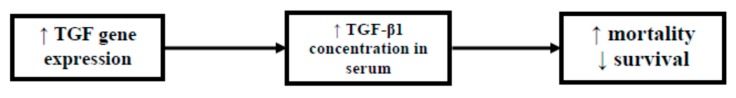
Relationship between changes in TGF gene expression and longevity.

The inflammatory process in a given tissue is the body’s response to a disruption of homeostasis. Cytokines, and among them TGF-β1 responsible for regulating the immune response, may play a special role in the ageing process. Carierri et al. studied a group of centenarians (aged 99–103) with a younger control group (aged 20–60) and found significant differences in genetic variability of the TGF-β1 gene with markedly higher plasma levels of the biologically active form of TGF-β1 in both men and women from the centenarian group. These findings point to a relationship between TGF-β1 genetic variability and longevity (Figure 2).

The authors additionally noted no relationship between the age-related increase in TGF-β1 levels and specific genetic variability (*p* > 0.05) [40]. At the same time, numerous studies have demonstrated links between TGF-β1 polymorphism and an increased risk of certain conditions, e.g., Alzheimer’s disease [41], osteoporosis [42], or hypertension [43]. 

Research into polymorphisms of IL-6, IL-10 and IFN-y carried out by Bosco et al. [41] in a group of 112 centenarians did not reveal a relationship between polymorphism and longevity in the study group. Further, Spoel et al. [44] sought to demonstrate a correlation between the serum levels of IGF-1, age and functional status in a group aged ninety years and older. Their findings in terms of IGF-1 levels failed to demonstrate any significant sex-specific differences (*p* = 0.98), while the concentration of IGFBP3 (insulin like growth factor binding protein 3) and IGF-1/IGFBP3 ratio demonstrated sex-dependent variability (respectively: *p* < 0.001; *p* < 0.001). At the same time, the authors found a positive correlation of the level of IGF-1 with longer life span and ability to function independently.

Longevity studies enable us to devise optimal strategies for planning care and satisfying the specific needs of the elderly population [45]. There are theories claiming that the ageing body accumulates damaged proteins and genetic material, which in consequence leads to apoptosis, chronic inflammation and age-related diseases. As a result, the changes caused by the diminished regenerative capacity and the accelerated accumulation of damage may overlap [46], hence strategies aimed at delaying the ageing process rely mainly on preventing or eliminating the damage done. There is no doubt that these mechanisms are determined by genetic, epigenetic and environmental factors. Gene variants correlated with longevity are known, and among them are those protein products involved in lipid metabolism and participate in nutrient sensing signaling pathways, such as the insulin/insulin-like growth factor (IGF-1) and mTOR pathways [47]. 

## 5. Conclusions

The aging process is extremely complex, but understanding it will allow real intervention in prolonging life. Selected biochemical parameters involved in the aging process determine changes in the human body. Research on processes related to aging and longevity will allow us to learn about the mechanisms responsible for them and enjoy these processes in good health. There is a need to conduct further research in the field of the occurrence of gender predispositions to significant dependencies in selected biochemical parameters.

## Figures and Tables

**Table 1:** Elements of the statistical description of the analysed variables according to the participants’ gender.

Variable		*n*	Mean	Min–Max	Q1–Q3	*p*
Age	Total	90	92.00	89–103	90–94	-
	Woman	69	92.00	89–103	90–94	0.228
	Man	21	92.00	89–96	90–93	
CRP [mg/L]	Total	90	7.31	0.74–46.29	2.96–12.88	-
	Woman	69	7.24	0.75–46.29	3.09–12.21	0.670
	Man	21	7.37	0.74–44.39	2.55–13.86	
Cortisol [μg/mL]	Total	89	0.16	0.04–0.46	0.1312–0.1871	-
	Woman	68	0.15	0.06–0.44	0.1312–0.1948	0.575
	Man	21	0.17	0.04–0.22	0.1205–0.1866	
FSH [mLU/mL]	Total	90	59.12	0.86–154.1	15.18–105.7	-
	Woman	69	69.33	0.86–154.1	15.62–113.1	0.189
	Man	21	36.87	3.86–150	14.9–84.1	
Insulin [µLU/mL]	Total	90	18.27	1.76–142.3	9.318–43.67	-
	Woman	69	17.52	1.76–117.5	8.641–41.25	0.338
	Man	21	19.98	2.32–142.3	10.98–47.42	
HGH [ng/mL]	Total	90	1.05	0.5–102	0.5062–2.097	-
	Woman	69	1.12	0.5–102	0.5062–2.795	0.067
	Man	21	0.80	0.5–4.833	0.5–1.046	
LH [ng/mL]	Total	90	8.47	0.32–39.54	2.282–14.85	-
	Woman	69	9.43	0.32–28.61	2.571–15.72	0.114
	Man	21	5.19	0.32–39.54	1.488–10.63	
Resistin [ng/mL]	Total	90	8.18	2.7–21.63	5.9724–10.8591	-
	Woman	69	8.30	2.8–21.63	6.5772–10.8591	0.465
	Man	21	7.46	2.7–16.42	4.8321–9.7629	
GHRH [pg/mL]	Total	88	137.55	13.42–2460	64.26–284.4	-
	Woman	67	170.50	13.42–2460	75.56–305	0.107
	Man	21	92.47	17.67–686.6	58.09–176.1	
Adiponectin [ng/mL]	Total	90	15,014.85	1727.01–48,422	8379.3–20,398	-
	Woman	69	15,066.00	3089.77–43,958	10,443.9–20,016.7	0.663
	Man	21	14,728.10	1727.01–48,422	5995.4–24,942.6	
GSH [Umol/L]	Total	90	6.72	4.51–44.7	6.074–9.179	-
	Woman	69	6.68	4.81–44.7	6.059–8.806	0.275
	Man	21	6.78	4.51–42.91	6.302–21.94	
GPx [ng/mL]	Total	90	41.28	13.2–247.7	36.98–63.14	-
	Woman	69	41.03	13.2–247	37.53–58.03	0.437
	Man	21	42.31	25.49–247.7	35.53–138.1	
CAT [ng/mL]	Total	90	56.27	30.3–511.3	48.32–77.48	-
	Woman	69	56.47	30.3–511.3	49.1–75.42	0.910
	Man	21	54.00	36.3–485.9	47.26–233.6	
GST [ng/mL]	Total	90	8.46	5.34–67.09	7.277–11.63	-
	Woman	69	8.37	5.34–65.03	7.277–11.28	0.399
	Man	21	8.78	6.02–67.09	7.474–31.71	
GSSG [µmol/L]	Total	90	2.76	1.46–15.44	2.448–3.902	-
	Woman	69	2.82	1.46–15.44	2.502–3.503	0.719
	Man	21	2.52	1.92–15.38	2.305–9.052	
R-GSSG [ng/mL]	Total	90	24.77	15.9–445.7	20.62–36.42	-
	Woman	69	24.20	15.9–396.4	20.62–33.59	0.532
	Man	21	25.00	15.9–445.7	20.62–109.8	
Homocysteine [nmol/mL]	Total	90	18.46	2.8–56.53	16.13–21.87	-
	Woman	69	18.42	2.8–56.53	16.13–21.43	0.616
	Man	21	18.68	12.4–54.16	16.13–33.23	
TNF-α [pg/mL]	Total	90	8.48	1.23–69.06	6.339–12.08	-
	Woman	69	8.85	1.23–69.06	6.78–12.46	0.071
	Man	21	7.22	2.40–17.72	5.02–11.32	
IL-1 α [pg/mL]	Total	88	102.00	1.12–102	102–102	-
	Woman	67	102.00	1.12–102	102–102	0.648
	Man	21	102.00	1.20–102	10.19–102	
IL-6 [pg/mL]	Total	90	27.84	3.13–668.8	13.2–85.72	-
	Woman	69	27.16	3.13–668.8	14.63–54.24	0.806
	Man	21	29.86	3.13–474.6	13.2–101	
SOD [ng/mL]	Total	90	37.44	22.1–133.1	33.09–49.06	-
	Woman	69	37.16	22.2–133.1	31.92–46.53	0.262
	Man	21	40.39	22.1–131.8	34.18–72.69	
IGF-1 [ng/mL]	Total	90	94.72	27.06–175.4	75.05–117.4	-
	Woman	69	95.05	27.06–175.4	78.83–115.6	0.305
	Man	21	92.40	35.93–156.6	63.23–117.4	
IL-1ß [pg/mL]	Total	90	101.00	1.07–1355	101–101	-
	Woman	69	101.00	1.07–1355	101–101	0.643
	Man	21	101.00	34.5–101	101–101	
IL-10 [pg/mL]	Total	89	3.11	1.6–631.7	1.6–22.98	-
	Woman	68	2.79	1.6–631.7	1.6–23.3	0.726
	Man	21	4.39	1.6–80.12	1.6–15.27	
TGF-ß1 [pg/mL]	Total	90	37,200.00	8565–12,6180	29,328–46,650	-
	Woman	69	36,750.00	8565–70,470	28,971–45,960	0.171
	Man	21	41,310.00	26,598–12,6180	31,920–50,850	
Fibrinogen [µg/mL]	Total	90	40.89	4.19–41.22	40.81–41.05	-
	Woman	69	40.89	5.31–41.22	40.81–41.05	0.507
	Man	21	40.89	4.185–41.14	40.81–41.05	

**Table 2:** The Spearman rank correlation analysis for insulin, including selected variables in the general group, men and women.

Variable		Cortisol	HGH	Adiponectin	IL-1α
		R(*p*)	R(*p*)	R(*p*)	R(*p*)
Insulin	Total	−0.333 (0.001)	−0.236 (0.025)	−0.247 (0.019)	0.176 (0.100)
	Woman	−0.238 (0.050)	−0.200 (0.100)	−0.195 (0.109)	0.283 (0.020)
	Man	−0.721 (0.000)	−0.376 (0.093)	−0.436 (0.048)	−0.083 (0.720)

HGH—human growth hormone; IL-1 α-interleukin-1 alpha.

**Table 3:** The Spearman rank correlation analysis for C-reactive protein (CRP) and HGH, including selected variables in the general group, men and women.

**Variable**		**Resistin**	**LH**	**FSH**	**GSSG**	**TNF-α**
		**R(** ***p*** **)**	**R(** ***p*** **)**	**R(** ***p*** **)**	**R(** ***p*** **)**	**R(** ***p*** **)**
CRP	Total	0.253 (0.016)	−0.343 (0.001)	−0.314 (0.003)	0.132 (0.214)	0.242 (0.022)
	Woman	0.475 (0.000)	−0.421 (0.000)	−0.443 (0.000)	−0.053 (0.664)	0.347 (0.003)
	Man	−0.382 (0.088)	−0.117 (0.613)	0.161 (0.486)	0.562 (0.008)	−0.114 (0.624)
**Variable**		**Resistin**	**LH**	**IGF−1**	**GHRH**	**Adiponectin**
		**R(*p*)**	**R(*p*)**	**R(*p*)**	**R(*p*)**	**R(*p*)**
HGH	Total	0.240 (0.023)	0.064 (0.547)	0.226 (0.032)	0.775 (0.000)	0.400 (0.000)
	Woman	0.215 (0.076)	−0.072 (0.558)	0.126 (0.301)	0.757 (0.000)	0.334 (0.005)
	Man	0.324 (0.152)	0.456 (0.038)	0.468 (0.032)	0.734 (0.000)	0.682 (0.001)

LH—luteinizing hormone; FSH—follicle-stimulating hormone; GSSG—oxidized glutathione; GHRH—growth hormone-releasing hormone.

**Table 4:** Spearman rank correlation analysis for luteinizing hormone (LH) and homocysteine, including selected variables in the general group, men and women.

**Variable**		**FSH**	**HGH**	**Resistin**	**GSH**	**GPx**	**CAT**	**GST**	**IL-6**	**SOD**	**IGF-1**
		**R(** ***p*** **)**	**R(** ***p*** **)**	**R(** ***p*** **)**	**R(** ***p*** **)**	**R(** ***p*** **)**	**R(** ***p*** **)**	**R(** ***p*** **)**	**R(** ***p*** **)**	**R(** ***p*** **)**	**R(** ***p*** **)**
LH	Total	0.866 (0.000)	0.064 (0.547)	−0.144 (0.175)	0.145 (0.174)	0.163 (0.124)	0.123 (0.249)	0.180 (0.089)	−0.184 (0.083)	0.129 (0.226)	0.239
											(0.023)
	Woman	0.838 (0.000)	−0.072 (0.558)	−0.265 (0.028)	0.297 (0.013)	0.344 (0.004)	0.257 (0.033)	0.269 (0.025)	−0.283 (0.019)	0.267 (0.026)	0.163
											(0.182)
	Man	0.839 (0.000)	0.456 (0.038)	0.226 (0.324)	−0.229 (0.318)	−0.329 (0.145)	−0.251 (0.272)	−0.005 (0.982)	0.098 (0.674)	−0.312 (0.168)	0.476
											(0.029)
**Variable**		**FSH**	**GHRH**	**GSH**	**GPx**	**CAT**	**GST**	**GSSG**	**R-GSSG**	**IL−6**	**SOD**
		**R(*p*)**	**R(*p*)**	**R(*p*)**	**R(*p*)**	**R(*p*)**	**R(*p*)**	**R(*p*)**	**R(*p*)**	**R(*p*)**	**R(*p*)**
Homocysteine	Total	0.088 (0.410)	−0.232 (0.030)	0.799 (0.000)	0.748 (0.000)	0.632 (0.000)	0.817 (0.000)	0.621 (0.000)	0.790 (0.000)	0.242 (0.021)	0.789 (0.000)
	Woman	0.247 (0.040)	−0.173 (0.161)	0.767 (0.000)	0.692 (0.000)	0.752 (0.000)	0.824 (0.000)	0.580 (0.000)	0.895 (0.000)	0.324 (0.007)	0.779 (0.000)
	Man	−0.303 (0.182)	−0.272 (0.234)	0.916 (0.000)	0.884 (0.000)	0.578 (0.000)	0.795 (0.000)	0.708 (0.000)	0.748 (0.000)	0.050 (0.829)	0.820 (0.000)

FSH—follicle-stimulating hormone; HGH—human growth hormone; GSH—glutathione; CAT—chloramphenicol acetyltransferase; GST—glutathione S-transferase; SOD—superoxide dismutase.

**Table 5:** The Spearman rank correlation analysis for growth hormone–releasing hormone (GHRH) and TNF-α, including selected variables in the general group, men and women.

**Variable**		**HGH**	**Adiponectin**	**GSH**	**R-GSSG**	**Homocysteine**	**IL−1α**
		**R(** ***p*** **)**	**R(** ***p*** **)**	**R(** ***p*** **)**	**R(** ***p*** **)**	**R(** ***p*** **)**	**R(** ***p*** **)**
GHRH	Total	0.775 (0.000)	0.314 (0.003)	−0.220 (0.040)	−0.224 (0.036)	−0.232 (0.030)	0.260 (0.014)
	Woman	0.757 (0.000)	0.286 (0.019)	−0.137 (0.269)	−0.172 (0.163)	−0.173 (0.161)	0.222 (0.072)
	Man	0.734 (0.000)	0.282 (0.216)	−0.256 (0.263)	−0.212 (0.357)	−0.272 (0.234)	0.279 (0.220)
**Variable**		**Resistin**	**GST**	**IL−6**	**IGF−1**	**IL−10**	**TGF−ß1**
		**R(*p*)**	**R(*p*)**	**R(*p*)**	**R(*p*)**	**R(*p*)**	**R(*p*)**
TNF-α	Total	0.397 (0.000)	0.137 (0.198)	0.017 (0.877)	0.301 (0.004)	0.216 (0.042)	−0.422 (0.000)
	Woman	0.401 (0.001)	0.242 (0.045)	0.181 (0.136)	0.332 (0.005)	0.234 (0.055)	−0.384 (0.001)
	Man	0.432 (0.051)	−0.093 (0.689)	−0.465 (0.034)	0.244 (0.287)	0.142 (0.539)	−0.500 (0.021)

HGH—human growth hormone; GSH—glutathione; R-GSSG—glutathione reductase; GST—glutathione S-transferase.

**Table 6:** The Spearman rank correlation analysis for IL-6, including selected variables in the general group, men and women.

Variable		LH	GST	R-GSSG	Homocysteine	TNF-α	SOD	IGF-1	IL-1ß
		R(*p*)	R(*p*)	R(*p*)	R(*p*)	R(*p*)	R(*p*)	R(*p*)	R(*p*)
IL-6	Total	−0.184 (0.083)	0.194 (0.066)	0.236 (0.025)	0.242 (0.021)	0.017 (0.877)	0.206 (0.052)	−0.062 (0.562)	−0.259 (0.014)
	Woman	−0.283 (0.019)	0.260 (0.031)	0.201 (0.383)	0.324 (0.007)	0.181 (0.136)	0.257 (0.033)	−0.025 (0.840)	−0.306 (0.010)
	Man	0.098 (0.674)	0.029 (0.900)	0.247 (0.041)	0.050 (0.829)	−0.465 (0.034)	0.097 (0.676)	−0.137 (0.553)	0.005 (0.982)

SOD—superoxide dismutase.

**Table 7:** The Spearman rank correlation analysis for follicle-stimulating hormone (FSH), glutathione S-transferase (GST), oxidized glutathione (GSSG) and glutathione reductase (R-GSSG), including selected variables in the general group, men and women.

**Variable**		**Resistin**	**GSH**	**GPx**	**CAT**	**GST**	**GSSG**	**R-GSSG**	**Homocysteine**	**SOD**	**IGF-1**	**IL-10**
		**R(** ***p*** **)**	**R(** ***p*** **)**	**R(** ***p*** **)**	**R(** ***p*** **)**	**R(** ***p*** **)**	**R(** ***p*** **)**	**R(** ***p*** **)**	**R(** ***p*** **)**	**R(** ***p)***	**R(** ***p*** **)**	**R(** ***p*** **)**
FSH	Total	−0.260 (0.013)	0.166 (0.119)	0.196 (0.064)	0.175 −0.100	0.226 (0.032)	0.219 (0.038)	0.117 (0.272)	0.088 (0.410)	0.155 (0.145)	0.196 (0.064)	−0.227 (0.033)
	Woman	−0.376 (0.001)	0.312 (0.009)	0.384 (0.001)	0.313 (0.009)	0.302 (0.012)	0.335 (0.005)	0.272 (0.024)	0.247 (0.040)	0.298 (0.013)	0.125 (0.306)	−0.242 (0.047)
	Man	0.135 (0.559)	−0.192 (0.404)	−0.252 (0.271)	−0.135 (0.559)	0.132 (0.567)	0.079 (0.733)	−0.216 (0.348)	−0.303 (0.182)	−0.257 (0.260)	0.504 (0.020)	0.003 (0.991)
**Variable**		**FSH**	**FSH**	**LH**	**GSH**	**GPx**	**CAT**	**GSSG**	**R−GSSG**	**Homocysteine**	**TNF-α**	**IL-6**
		**R(*p*)**	**R(*p*)**	**R(*p*)**	**R(*p*)**	**R(*p*)**	**R(*p*)**	**R(*p*)**	**R(*p*)**	**R(*p*)**	**R(*p*)**	**R(*p*)**
GST	Total	0.226 (0.032)	0.180 (0.089)	0.898 (0.000)	0.861 (0.000)	0.768 (0.000)	0.782 (0.000)	0.812 (0.000)	0.817 (0.000)	0.137 (0.198)	0.194 (0.066)	0.835 (0.000)
	Woman	0.302 (0.012)	0.269 (0.025)	0.900 (0.000)	0.871 (0.000)	0.794 (0.000)	0.772 (0.000)	0.821 (0.000)	0.824 (0.000)	0.242 (0.045)	0.260 (0.031)	0.873 (0.000)
	Man	0.132 (0.567)	−0.005 (0.982)	0.884 (0.000)	0.817 (0.000)	0.754 (0.000)	0.826 (0.000)	0.797 (0.000)	0.795 (0.000)	−0.093 (0.689)	0.029 (0.900)	0.722 (0.000)
**Variable**		**FSH**	**FSH**	**GSH**	**GPx**	**CAT**	**GST**	**R−GSSG**	**Homocysteine**	**SOD**	**IGF-1**	**TGF-ß1**
		**R(*p*)**	**R(*p*)**	**R(*p*)**	**R(*p*)**	**R(*p*)**	**R(*p*)**	**R(*p*)**	**R(*p*)**	**R(*p*)**	**R(*p*)**	**R(*p*)**
GSSG	Total	0.219 (0.038)	0.750 (0.000)	0.794 (0.000)	0.850 (0.000)	0.782 (0.000)	0.623 (0.000)	0.621 (0.000)	0.732 (0.000)	0.129 (0.226)	−0.149 (0.162)	0.232 (0.028)
	Woman	0.335 (0.005)	0.738 (0.000)	0.786 (0.000)	0.842 (0.000)	0.772 (0.000)	0.572 (0.000)	0.580 (0.000)	0.723 (0.000)	0.286 (0.017)	−0.239 (0.048)	0.186 (0.125)
	Man	0.079 (0.733)	0.786 (0.000)	0.845 (0.000)	0.833 (0.000)	0.826 (0.000)	0.774 (0.000)	0.708 (0.000)	0.735 (0.000)	−0.125 (0.590)	0.105 (0.652)	0.418 (0.059)
**Variable**		**FSH**	**FSH**	**GHRH**	**GSH**	**GPx**	**CAT**	**GST**	**GSSG**	**Homocysteine**	**IL-6**	**SOD**
		**R(*p*)**	**R(*p*)**	**R(*p*)**	**R(*p*)**	**R(*p*)**	**R(*p*)**	**R(*p*)**	**R(*p*)**	**R(*p*)**	**R(*p*)**	**R(*p*)**
R-GSSG	Total	0.117 (0.272)	−0.224 (0.036)	0.813 (0.000)	0.780 (0.000)	0.700 (0.000)	0.812 (0.000)	0.623 (0.000)	0.790 (0.000)	0.236 (0.025)	0.766 (0.000)	−0.122 (0.256)
	Woman	0.272 (0.024)	−0.172 (0.163)	0.777 (0.000)	0.756 (0.000)	0.857 (0.000)	0.821 (0.000)	0.572 (0.000)	0.895 (0.000)	0.201 (0.383)	0.852 (0.000)	0.306 (0.177)
	Man	−0.216 (0.348)	−0.212 (0.357)	0.913 (0.000)	0.829 (0.000)	0.645 (0.000)	0.797 (0.000)	0.774 (0.000)	0.748 (0.000)	0.247 (0.041)	0.728 (0.000)	−0.254 (0.037)
